# Extraversion level predicts perceived benefits from social resources and tool use

**DOI:** 10.1038/s41598-021-91298-w

**Published:** 2021-06-10

**Authors:** Vincent Murday, Kévin Campos-Moinier, François Osiurak, Lionel Brunel

**Affiliations:** 1 Laboratoire Epsylon EA 4556, Université Montpellier 3, Montpellier, France; 2 Laboratoire EMC, Université Lyon 2, Lyon, France

**Keywords:** Human behaviour, Behavioural ecology

## Abstract

Social baseline theory states that there are differences in how humans integrate social resources into their economy of action when they face environmental demands. However, although several authors suggested that extraversion may be an indicator of the social baseline, no study has demonstrated it. The present study aims to test this hypothesis and, in particular, examines whether extraversion is a specific indicator of the social baseline. In two experiments, participants were asked to move rolls either alone (with their hands), or with the help of a social resource (Experiment 1), or a tool (Experiment 2). Results showed that extraversion predicted the choice to use both types of resource. Specifically, the more participants were extraverted, the more they tended to consider the use of the social resource or the tool as beneficial. We argue that these results indicate that extraversion is not specifically an indicator of the social baseline, but rather an indicator of how individuals integrate technical and social resources into their economy of action. In addition, this study encourages future research endeavors to define what constitutes a resource and how it could fit into the Social Baseline Theory.

## Introduction

Imagine sitting in your living room, watching television with a friend. The movie ends, you have finished your meal and it’s time to clear your coffee table. Two options are available: (1) you can make several trips, moving things with both your hands; or (2) you can ask your friend for help. We can assume that, depending on the quantity of objects you have to move, asking your friend would be a benefit, and a way to reduce the cost required to clear the table. However, what is the threshold quantity to move to estimate the help of your friend as beneficial? Specifically, is the threshold quantity stable across individuals, or does a dispositional trait (e.g., personality) modulate such estimation?

The principle of economy of action states that energy must be conserved over time^1^. This principle is clearly demonstrated in the field of behavioral ecology, which highlights that animals are sensitive to the cost–benefit ratio^2–4^. For instance, worker bees are sensitive to the cost–benefit ratio associated with harvested nectar. As the weight of the nectar increases the energy cost of the flight, bees return to the hive with a quantity of nectar that is lower than the maximum they could carry, to optimize the harvest^3^. As for humans, we find this economy of action in the fields of motor movement^5,6^ or perceptual activity^7–9^, among others. For instance, individuals choose paths that minimize the number of steps when they navigate an intersection^6^, and they tend to view hills as steeper if they are fatigued, less physically fit, or are carrying a heavy backpack^7^. These variations in visual perception would aim to regulate the adoption of appropriate actions, on the basis of anticipated costs and benefits^10^.

To economize actions, it is necessary to begin by determining a baseline of resources that organisms seek to maintain^11^. In other words, organisms need a reference level, in order to be able to evaluate if a costly action must be performed, depending on the benefits it may confer. In humans, this baseline takes into account both physiological resources (e.g., glucose) and social resources (e.g., friends and family). Earlier experimental results are consistent with this idea. For instance, individuals estimate a box to be lighter when they expect a partner to help^12^, or a hill to be less steep when they are in the company of a friend than when they are alone^13^.

Social baseline theory (SBT) argues that the human brain assumes that social resources are construed as bioenergetic resources, and as a way to economize behavior^14,15^. More precisely, “[the] social baseline indicates the degree to which an individual incorporates others in their network of social resources”^11^—and the higher the baseline is, the more an individual incorporates social resources (SR) into their economy of action. For instance, someone with a high social baseline will tend to be interdependent, distributing the cost of his or her action across the social environment while, conversely, a person with a low social baseline will tend to be independent.

Some authors^11,16^ suggest that the social baseline is a function of personality traits linked to social interaction, such as extraversion. Extraversion reflects our general tendency to seek out others and interact with them^17–20^. Extraverts tend to have a larger social network, and appear to have more contacts with the members of this network than introverts^21^. In addition, they tend to seek more social support than introverts^22–24^. For instance, Amirkhan et al.^23^ showed that extraversion is positively correlated with help-seeking behavior. In their experiment, participants were asked to solve insolvable anagrams. The results showed the greater the degree of extraversion, the more quickly the person asked help from a SR posted outside the room. Taken together, these results suggest that extraverts are more likely to be interdependent than introverts, which, in turn, indicates that the social baseline could vary as a function of extraversion. Thus, the more an individual is extraverted, the more the person should evaluate SR as a way to reduce the cost of action (i.e., as a benefit for action). However, since the hypothesis was initially put forward by Gross and Proffitt in 2013, to date, no study has investigated it.

Thus, the present study aims to test whether extraversion is an indicator of the social baseline, i.e., the way that individuals incorporate SR into their economy of action. To this end, we adopted an experimental design similar to the one used in a previous study^25^ that investigated how individuals perceive the benefits of tools. The latter study aimed to establish a “baseline” for each individual—i.e., the moment from which the individual considered the use of the tool as being a benefit for action, as a function of the costs incurred by its use. The authors manipulated the cost of action, and observed whether participants decided to use the tool. Specifically, participants were asked to move different quantities of toilet tissue rolls, either directly with their hands (two at a time), or with a tool (four at a time). However, if they used the tool, they had to go and collect it and bring it back, which required an additional movement. Our study adopted the same design, except that we replaced the tool with a SR, and measured extraversion with the Big Five Inventory (BFI). We hypothesized that Extraversion would predict the way participants use the SR. More precisely, we predicted that the more an individual would be extraverted, the more the person would choose to use the SR.

## Experiment 1

### Participants

The sample consisted of 36 participants (*M*_age_ = 22.72, *SD*_age_ = 1.91), which were recruited during the autumn semester at the Saint-Charles cafeteria of the University Paul Valery, France. All participants completed an informed consent form and reported being right-handed, had normal or corrected-to-normal visual acuity, and normal motricity. We chose a sample composed exclusively of women due to the existence of differences between men and women in the use of SR^26^. The aim was also to avoid a possible effect of sexism (e.g., men might not use the SR because it is a woman)^27,28^. The sample size was determined with G*Power^29^. A hypothesized effect size of − 0.43 was chosen, based on a previous study^23^; a corresponding power analysis (effect size r = − 0.43, α = 0.05, power = 0.80) resulted in an estimation of 32 participants. The final sample size (36) was chosen to meet methodological needs. The study was conducted according to the Declaration of Helsinki and were reviewed and approved by the Scientific and Ethics Committee of Epsylon Laboratory EA4556, University Paul Valéry of Montpellier.

### Materials and stimuli

**Figure 1:**
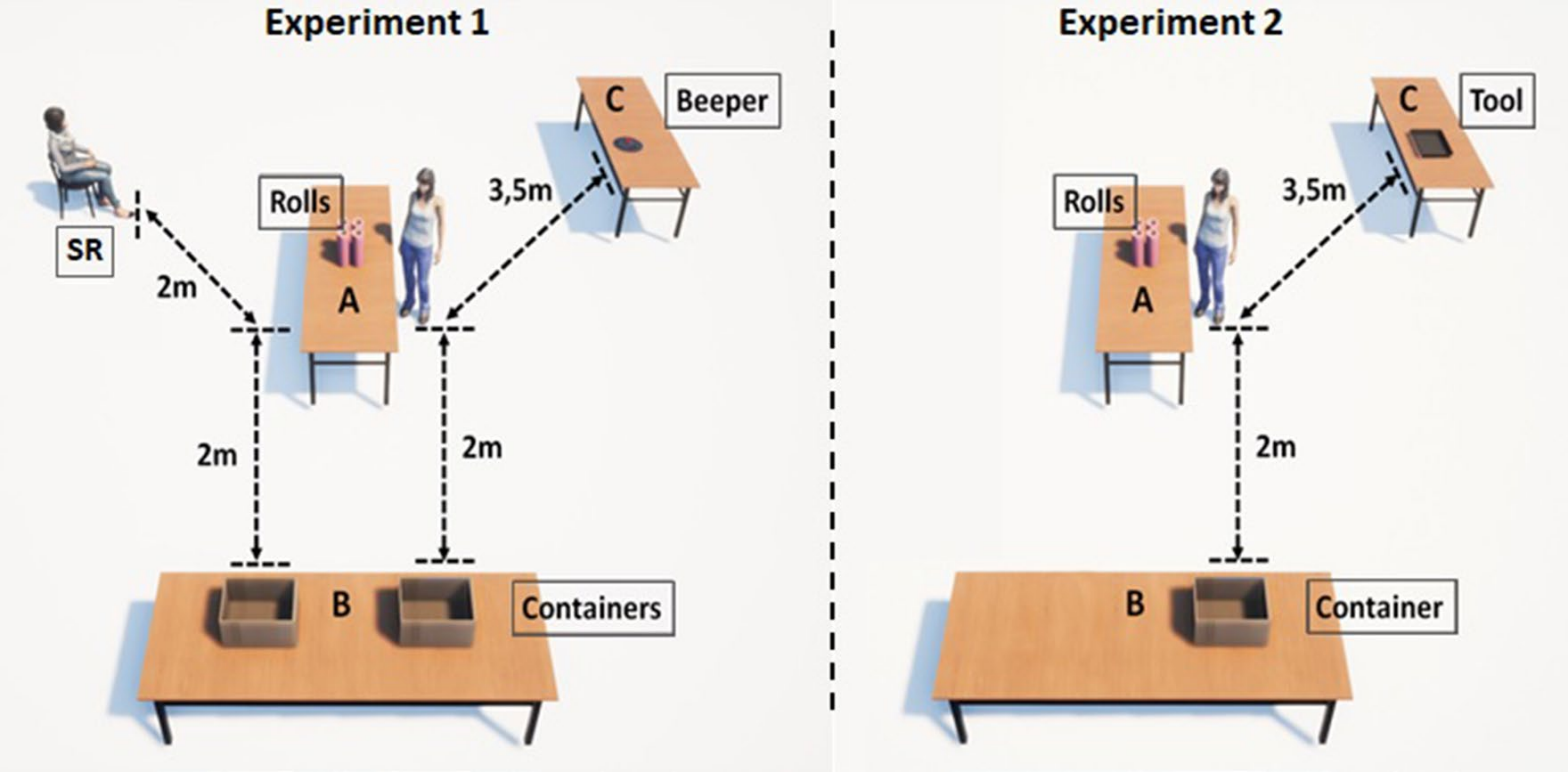
Schematic representation of the design used in Experiments 1 and 2.

A schematic representation of the experimental design is presented in Fig. 1 left-hand side. Three, rectangular tables were placed some distance apart from each other. In the real action task, toilet tissue rolls (either 16 or 8) were positioned in rows on Table A, with each row containing four rolls (e.g., for eight rolls, there were two rows of four rolls). The aim was to use objects that can be grasped with hands and light enough to limit the impact of the weight. Participants were asked to move the rolls from Table A to Table B and to put them into containers disposed on Table B. There were two containers (height: 30 cm, width: 55 cm, depth: 35 cm) in order to avoid interference between the participant and the SR during the real action task. A beeper was placed on Table C. Participants had to press on this beeper to ask for help from the SR. For all participants, the SR was a 23-year-old woman student who sat on a chair 2 m from Table A. The distances AB and AC were 2 m and 3.5 m, respectively.

**Figure 2:**
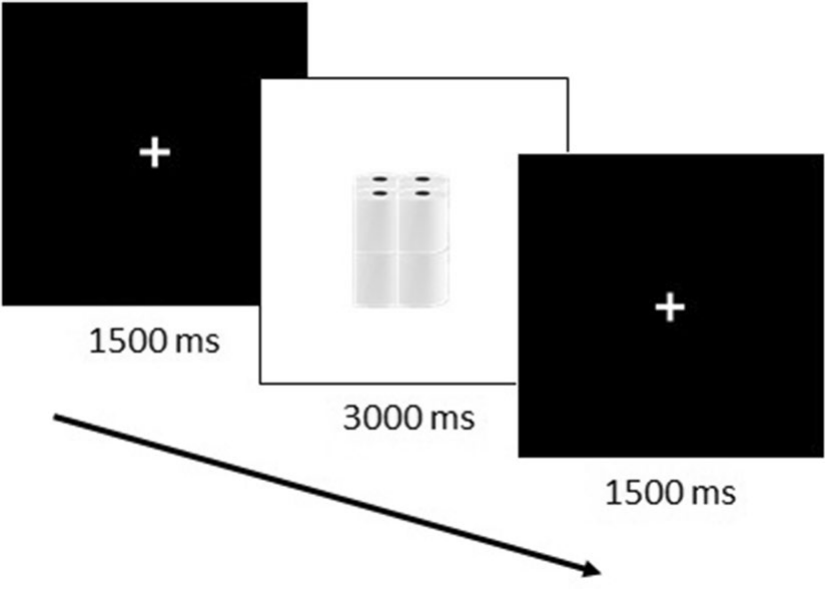
Experimental design for decision task in Experiment 1 and 2.

The decision task (Fig. 2) was computer-based, and developed with OpenSesame software^30^. Participants were seated at a table located 3 m from Table A, in front of a monitor and keyboard (screen size: 14-inch; distance participant-screen: 75 cm; distance participant-keyboard: 30 cm). Photographs showing a quantity of 4 (Q4), 8 (Q8), 12 (Q12), 16 (Q16), 20 (Q20), and 24 (Q24) rolls were displayed on the screen.

Personality traits were assessed by a self-report questionnaire: the Big Five Inventory (BFI)^31^ translated and validated in a French population^32^. This questionnaire is based on the “Big Five”, the five dimensions consensual model of adult personality^33^. The Big Five dimensions are: Conscientiousness, Agreeableness, Neuroticism, Openness to experience, and Extraversion.

### Procedure

Participants were informed that the experiment was composed of four phases. In the first phase they would be asked to fill out the BFI. In a second phase called the real action task, they would have to move different quantities of rolls with their hands, or with the help of a SR. In the third phase, the decision task, they could decide to use the SR or not depending on the actions performed in the second phase. Finally, they were told that in the last, fourth, phase they would be asked to move different quantities of rolls depending on the choices they had made in the decision task (third phase). Here, the aim was to raise the participant’s awareness of the impact and importance of the decision task, but this fourth phase was never actually done.

After the BFI completion, participants began phase two. Here, they were asked to physically move rolls from Table A at normal speed (i.e., without any time constraint) and place them in the container on Table B. This scenario was designed to reflect a real action task of storing purchases after returning from the supermarket. The experimenter asked participants to perform four trials: two in the Hands condition (quantities 8 and 16), and two in the SR condition (quantities 8 and 16). In the Hands condition, participants had to move all the rolls from Table A to Table B and only two at a time. Once the task was completed, they had to return to Table A. In the SR condition, participants had to move all rolls with the help of the SR. Here, they had to move from Table A, go to Table C to press the beeper (which sounded instantly), then return to Table A and move rolls with their hands, two at a time, to the container on Table B. Simultaneously, the co-actor got up from her chair and went to Table A to help participants to move the rolls. The co-actor also moved two rolls at a time, in synchrony with participants. Thus, participants and the co-actor moved four rolls, at the same time, with their hands. When the task was completed, participants had to go back to Table A, then Table C, press the beeper a second time, and finally return to Table A. The variables Quantity of rolls (8 vs. 16) and Condition (Hands vs. SR) were counterbalanced between participants, leading to the four following orders: (1) Hands/Q8, SR/Q16, Hands/Q16, SR/Q8, (2) Hands/Q16, SR/Q8, Hands/Q8, SR/Q16, (3), SR/Q8, Hands/Q16, SR/Q16, Hands/Q8, (4) SR/Q16, Hands/Q8, SR/Q8, Hands/Q16.

As mentioned above, decision task (third phase) was computer based. Each trial began with a white fixation cue presented in the center of a black screen displayed for 1500 ms. The fixation cue was immediately followed by a photograph showing a quantity of rolls (Q4 vs. Q8 vs. Q12 vs. Q16 vs. Q20 vs. Q24) displayed for 3000 ms on the screen. Participants had to evaluate 20 times each Quantity of Rolls (6 Quantity of Rolls × 20 Blocks), making a total of 120 photographs to be evaluated. They were asked to respond as rapidly as they could before the photograph disappeared from the screen. In case they did not respond to a trial in the allotted time, the trial was presented later in its corresponding block. Participants were asked to press the ‘a’ key with their left index finger, or the ‘p’ key with their right index finger if they considered being faster to move the rolls with the SR or by hands. The response assigned to each key was counterbalanced across participants and the quantity of rolls was fully randomized. Response times and decisions were collected for each of the 120 photographs. Participants were informed that after the decision task, the software would randomly choose 12 photographs, and in a second real action task (i.e., fourth phase), they would have to move these quantities of rolls depending on the choices they had previously made. Moreover, they were informed that the SR was at their complete disposal and they were invited to choose its use according to their own estimate. In fact, once the decision task was over, they were informed that they would not have to complete the fourth phase. Upon recruitment, they were told that the experiment would take 40 min (i.e., enough time to also complete the fourth phase). However, the actual duration of the experiment was 30 min (the time to complete the BFI, the real action task and the decision task).

### Results

Raw responses were converted to a binary response, based on the participant’s choice (0 for Hands, 1 for SR). These data were then fitted locally using the ModelFree software package^34^, giving a point of subjective equality (PSE-SR) for each participant. Specifically, the PSE-SR indicates the quantity of rolls for which the participant considered the use of their hands, or the SR as equivalent. After a visual inspection of the psychometric curve, data of four participants were excluded from the analysis because these participants had particularly flat curve (i.e., slope = 0). Then, we used the Shapiro test to test the normality of distributions of all dependent variables. PSE-SR, Neuroticism, Openness to experience, Conscientiousness, and Extraversion were identified to be normally distributed (all *W* [0.940, 0.979], and *p* [0.070, 0.78]), while Agreeableness was not (*W* = 0.933, *p* = 0.048). Hence, the use of parametric statistical tests was proscribed, and the analysis of the correlation matrix was performed with the Spearman non-parametric method.

**Figure 3:**
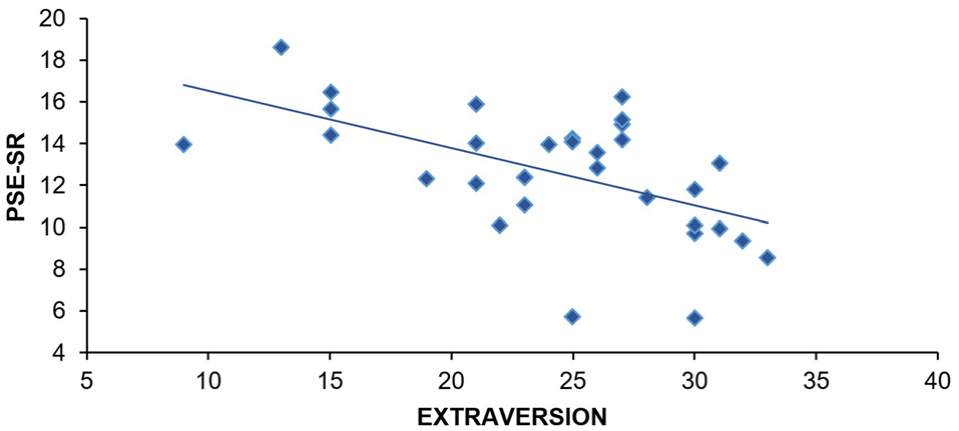
Correlation between Extraversion and PSE-SR found in Experiment 1.

Correlations matrix between the five personality factors, and the PSE-SR score are given in Table 1, which highlights a correlation between Extraversion and PSE-SR (Fig. 3). Data were also examined by estimating Bayes factors comparing fit under the null hypothesis (PSE-SR is not a function of personality trait), and the alternative hypothesis (PSE-SR is a function of personality trait). Bayes factors were, respectively, *BF*_*01*_ = 3.481 for Neuroticism, and *BF*_01_ = 3.728 for Conscientiousness. These results confirm the null hypothesis, as Bayes factors are above three^35^. Bayes factors were, respectively, *BF*_01_ = 1.550 for Openness to experience, and *BF*_01_ = 1.117 for Agreeableness. As these results do not support either the null nor the alternative hypothesis, a multiple regression was conducted to test whether Extraversion, Agreeableness, and Openness to experience predicted PSE-SR.

**Table 1:** Spearman correlation coefficients for PSE-SR and personality factors, and among personality factors for Experiment 1.

	PSE-SR	Openness	Conscientiousness	Extraversion	Agreeableness
Openness 95% CI	− .256 [− .555, .102]				
Conscientiousness 95% CI	.204 [− .156, .516]	.153 [− .206, .477]			
Extraversion 95% CI	− .534** [− .744, -.227]	.213 [− .147, .523]	− .082 [− .419, .274]		
Agreeableness 95% CI	− .212 [− .522, .147]	.131 [− .228, .459]	.097 [− .261, .431]	.217 [− .142, .526]	
Neuroticism 95% CI	.057 [− .298, .398]	− .141 [− .467, .218]	− .167 [− .487, .193]	− .150 [− .474, .209]	− .497** [− .721,− .180]

PSE-SR is the quantity of rolls for which the participant considered the use of the SR and their hands as equivalent. 95% CI is the confidence interval. Significance levels are indicated as follows: *p < .05, **p < .01, ***p < .001.

The multiple regression showed that these three factors explain a significant amount of variance in the value of PSE-SR, *F*(3,31) = 5.087, *p* = 0.006, *R*^2^_*Adjusted*_ = 0.283*.* Specifically, Agreeableness (*β* =  − 0.141, *t* =  − 0.882, *p* = 0.385, 95% CI = [− 0.258, 0.103]), and Openness to experience (*β* =  − 0.159, *t* =  − 1.023, *p* = 0.314, 95% CI = [− 0.237, 0.079]) did not significantly predict the value of PSE-SR. However, Extraversion did (*β* =  − 0.480, *t* =  − 2.958, *p* = 0.006, 95% CI = [− 0.402, − 0.073]).

### Discussion

The key finding of Experiment 1 is the significant negative linear relationship between Extraversion and PSE-SR. This result supports our hypothesis, and shows that participants’ Extraversion level predicts the use of the SR. The more participants are extraverted, the more they perceive the SR as a benefit. This is consistent with the work of Amirkhan et al.^23^, which shows that extraverts seek help much more quickly than introverts. They also extend the results obtained by Schnall et al.^13^ and Doerffeld et al.^12^, namely that there are individual differences in how the benefits of a SR are perceived: the more participants are extraverted, the more they tend to incorporate SR as a benefit into their economy of action. However, a new question arises: does Extraversion only influence how people perceive the benefits of SR, or does it extend to other external resources? To investigate this question, we replicated the second experiment reported in Osiurak et al.^25^ (i.e. we replaced the SR with a tool) and added the BFI.

## Experiment 2

Experiment 2 investigated how individuals estimate the benefits of a tool depending on their Extraversion level, measured with the BFI. The main objective was to investigate whether Extraversion only influences how individuals perceive the benefits of SR, or if this effect extends to other types of resources, such as a tool. If it only influences SR, no relation should be found between Extraversion and the decision to use the tool, and vice versa.

### Participants

To the best of our knowledge, no study reported differences between men and women in tool use. Hence, the sample of this experiment consisted of 36 participants (*M*_*age*_ = 23.17, *SD*_*age*_ = 2.46) of both sexes (21 women and 15 men). As in Experiment 1, participants were recruited during the autumn semester at the Saint-Charles cafeteria of the University Paul Valery, France. They completed an informed an informed consent form and reported being right-handed, with normal or corrected-to-normal visual acuity, and normal motricity. The study was conducted according to the Declaration of Helsinki and were reviewed and approved by the Scientific and Ethics Committee of Epsylon Laboratory EA4556, University Paul Valéry of Montpellier.

### Materials and procedure

Materials and procedure were basically the same as those used in Experiment 1 (Fig. 1, right-hand side). However, the SR was replaced by a tool placed on Table C. This tool was a tray large enough (height: 2 cm, width: 34.5 cm, depth: 26.5 cm) to allow participants to move four rolls at a time and light enough (200 g) to limit the impact of the weight. Thus, in the Tool condition, participants could move rolls four at a time with the tray. The task objective was the same as the Experiment 1: move all the rolls at normal speed (i.e., without any time constraint) from Table A to Table B. To complete this objective, participants started from Table A, they had to collect the tray from Table C, return to Table A to collect the rolls, and move them to Table B. When the task was completed, they had to return to Table A, then return the tray to Table C, and return to Table A.

### Results

As in Experiment 1, raw responses were converted to a binary response based on the participant’s choice (0 for Hands, 1 for Tool). These data were then fitted locally using the ModelFree software package^34^, giving a point of subjective equality (PSE-T) for each participant. The PSE-T indicates the quantity of rolls for which the participant considered the use of their hands, or the Tool as equivalent. After a visual inspection of the psychometric curve, data of one participant were excluded from the analysis because this participant had a flat curve (i.e., slope = 0). Here again, we used the Shapiro test to examine the normality of distributions of all dependent variables. All variables were normally distributed (all *W* [0.950, 0.983], and *p* ∈ [0.114, 0.866]). Hence, the analysis of the correlation matrix was performed with the parametric Pearson method.

Correlations between the five personality factors, and the PSE-T score are given in Table 2, which highlights that no correlations were found. Then, as in Experiment 1, data were examined by estimating Bayes factors, and comparing fit under the null hypothesis (personality trait does not predict the PSE-T), and the alternative hypothesis (personality trait does predict the PSE-T). Bayes factors were, respectively, *BF*_01_ = 4.762 for Openness to experience, *BF*_01_ = 4.762 for Agreeableness, and *BF*_01_ = 3.160 for Neuroticism. All of these results confirm the null hypothesis, as Bayes factors are above three^35^. However, Bayes factors were *BF*_01_ = 1.624 for Conscientiousness, and *BF*_01_ = 1.428 for Extraversion, which refer to a non-conclusive result. Therefore, a multiple regression was conducted to test whether Extraversion and Conscientiousness predicted the PSE-T. This indicated that these two factors explain a significant amount of variance in the value of PSE-T, *F*(2,34) = 3.352, *p* = 0.048, *R*^*2*^_*Adjusted*_ = 0.122*.* More precisely, the analysis showed that Conscientiousness did not significantly predict PSE-T (*β* = 0.320, *t* = 1.955, *p* = 0.059, 95% CI = [− 0.007, 0.359]), while Extraversion did (*β* =  − 0.333, *t* =  − 2.032, *p* = 0.050, 95% CI = [− 0.339, 0]).

**Table 2:** Pearson correlation coefficients for PSE-T and personality factors, and among personality factors in Experiment 2.

	PSE-T	Openness	Conscientiousness	Extraversion	Agreeableness
Openness 95% CI	− .012 [− .343, .323]				
Conscientiousness 95% CI	.258 [− .083, .544]	.103 [− .238, .422]			
Extraversion 95% CI	− .273 [− .556, .066]	− .088 [− .410, .252]	.187 [− .156, .489]		
Agreeableness 95% CI	− .021 [− .352, .314]	.011 [− .343, .323]	.249 [− .092, .538]	.021 [− .314, .352]	
Neuroticism 95% CI	.161 [− .182, .469]	− .256 [− .543, .084]	− .208 [− .506, .135]	− .172 [− .478, .171]	− .257 [− .544, .083]

PSE-T is the quantity of rolls for which the participant considered the use of the tool and their hands as equivalent. 95% CI corresponds to the confidence interval. Significance levels are indicated as follows: *p < .05, **p < .01, ***p < .001.

The PSE-T was submitted to a one-way ANOVA, with Sex as a between-participants factor. The analysis revealed no significant effect of Sex on PSE-T, *F*(1, 33) = 1.121, *p* = 0.297, ${\eta }_{\rho }^{2}$ = 0.033.

### Additional analyses

In order to test whether the samples of Experiments 1 and 2 originate from the same population, we compared our data for all personality traits. The analysis revealed no difference in Conscientiousness (*t*(65) = 0.797, *p* = 0.428, Cohen’s *d* = 0.195, 95% CI for Cohen’s *d* = [− 0.286, 0.675]), Extraversion (*t*(65) = − 1.677, *p* = 0.098, Cohen’s *d* = − 0.410, 95% CI for Cohen’s *d* = [− 0.893, 0.076], Neuroticism (*t*(65) = 1.495, *p* = 0.140, Cohen’s *d* = 0.366, 95% CI for Cohen’s *d* = [− 0.119, 0.848]. However we found a difference in Openness (*t*(65) =  − 2.611, *p* = 0.011, Cohen’s *d* = − 0.639, 95% CI for Cohen’s *d* = [− 1.128, − 0.145] and in Agreeableness (*W* = 724, *p* = 0.040, Rank-Biserial Correlation = 0.293, 95% CI for Rank-Biserial Correlation = [0.023, 0.523].

Additionally, data from Experiments 1 and 2 were entered in an ANCOVA with Experiment (Social vs. Tool) as a between-participant factor, Extraversion as covariate and PSE (PSE-SR and PSE-T) as the dependent variable. The analysis revealed no significant interaction between Extraversion and Experiment, *F*(1, 63) = 1.398, *p* = 0.241, ${\eta }_{\rho }^{2}$ = 0.017. However, it revealed significant main effects of Extraversion, *F*(1, 63) = 12.653, *p* < 0.001, ${\eta }_{\rho }^{2}$ = 0.151 and Experiment, *F*(1, 63) = 6.989, *p* = 0.011, ${\eta }_{\rho }^{2}$ = 0.083). Specifically, PSE-SR (M = 12.619, SD = 2.945, N = 32) was greater than PSE-T (M = 10.307, SD = 3.034, N = 35).

### Discussion

Extending the result obtained in Experiment 1, we found that Extraversion predicts the PSE-T. The more participants were extraverted, the more they used the tool. This result is in line with the hypothesis that the relationship between Extraversion and the perceived benefits of a resource is not limited to SR, and can be extended to technical resources, such as tools. Additionally, unlike Experiment 1, we found a trend effect of Conscientiousness on PSE-T. This effect could be explained by the fact that conscientious people tend to perform a task well and be efficient^36^. Therefore, it is possible that the more participants were conscientious, the less they tended to overestimate the benefits provide by the tool. Interestingly, the effect of Conscientiousness opposes the effect of Extraversion on PSE-T. Thus, participants who scored low in Conscientiousness and high in Extraversion perceived the benefits provided by the tool as being superior to participants who scored high in Conscientiousness and low in Extraversion. Moreover, no effect of Sex on PSE-T was found suggesting that men and women perceived the benefits of the tool equally. Overall, our findings extend the results obtained by Osiurak et al.^25^ and shows there are inter-individual differences in the way people estimate the benefits of tools which may be partly explained by personality traits. Finally, the effect of Support on PSE found in the additional analysis indicates that participants estimated the benefits of the SR and the tool differently. Specifically, they estimated the SR less beneficial than the tool, suggesting additional costs to use SR.

## General discussion

The main objective of this study was to investigate whether Extraversion could be an indicator of the social baseline, i.e., how individuals incorporate social resource (SR) into their economy of action. In the first experiment, we asked participants to estimate whether a SR was more beneficial than working alone, as a function of the quantity of rolls to be moved. In the second experiment, we replaced the SR by a tool. Here, the aim was to investigate if Extraversion only influences how individuals perceive the benefits of a SR, or whether it extends to technical resources, such as a tool. Our results indicated that Extraversion predicted the perceived benefits of both SR (Experiment 1) and tool use (Experiment 2). We also found that the PSE differed as a function of the type of support. Specifically, participants considered the tool to be more beneficial than the SR.

First, we suggest that participants’ estimations reflect how they incorporate the SR and the tool into the anticipated costs and benefits of their action. The more they see these resources as a benefit, the more they are open to incorporate them into their economy of action. This is a key assumption in the Social Baseline Theory (SBT), where SR are considered as bio-energetic resources, and a way to share the load^15^. We argue that, to some extent, technical resources such as tools can be viewed in the same way. Previous work argued that tools can be considered as a way to extend sensorimotor capacities^37^, thus modifying the perceived cost of action^25,38^.

Second, our finding showed that the more participants were extraverted, the more they estimated the SR as a benefit. This result is in line with our main hypothesis that the more individuals are extraverted, the more they incorporate SR into their economy of action. Specifically, it suggests that Extraversion could be an indicator of the social baseline^11,16^; extraverts would have a higher social baseline than introverts, they would be more interdependent and tend to share the load on their social environment. However, the link between Extraversion and tool use identified in Experiment 2 indicates that Extraversion is not a specific indicator of the social baseline, but, rather, an indicator of how individuals incorporate the resources (social and technical) present in their environment into their economy of action. These results seem consistent with the assertion that Extraversion is linked to an underlying sensitivity to reward^39^. According to the latter approach, Extraversion reflect how individuals are attracted to the rewards offered by situations. Thus, extraverts are not only more inclined to engage in social situations, but they are also more inclined to engage in beneficial situations^40^.

Another finding is that SR was estimated as offering less benefit than the tool. This is well illustrated by the observed difference in PSE between both experiments, but also with regard to the analysis of decision times (see Additional material). Contrary to the results obtained in Experiment 2, the decision times between Q12 and Q16 were not significantly different in Experiment 1. This suggests that participants were more hesitant about the benefits they could get from using the SR at Q12 compared to the use of the tool. On the one hand, this result could reflect additional costs associated with interpersonal coordination or requesting assistance. Thus, by increasing the weight of the objects to be moved (e.g., using rocks instead of rolls), this potential cost could become negligible compared to the benefits it would provide. However, unlike the SR, the tool used in our study did not allow the weight distribution. Consequently, an increase in the weight of the objects to be moved could also impair the perceived benefits of the tool. On the other hand, the perceived benefit of the SR could also have been modulated by the fact that it was a stranger. Indeed, according to SBT, load sharing is facilitated by trust and interdependence^15^. Thus, reinforcing the social proximity between participants and the SR (e.g., choosing a friend or a family member) could have increase the perception of SR as a means to distribute the costs of the action. This is consistent with Schnall et al.^13^, who found that the effect of SR on visual perception is mediated by interpersonal proximity. It is also consistent with Coan et al.^41^, who found that threat-related neural activity decreases in presence of a SR and this as a function of the quality of the relationship. However, although social proximity facilitates load sharing, this occurs mainly because the SR represents a reliable way to minimize costs in a given situation (e.g., depending on the constraints, risks or opportunities). In other words, the more individuals would be confident about the benefits that a SR can provide (e.g., based on the efficacity, availability and responsiveness), the more they would tend to share the load depending on their Social Baseline^14^. In our study, we believe that this reliability was perceived by participants, particularly through the real action task and the experimental instructions (i.e., the fact that the SR was at their disposal).

In addition, our results raise the question of whether there is a real distinction between social and technical resources –or whether differences in durability, reliability, and predictability influence how these resources are integrated into the economy of action of individuals. Both SR^15^ and material objects^42^ have been found to be perceived as an “expansion of the self”. Indeed, emotional investment in a person or an object can be so great that their loss results in real distress. On the other hand, we can objectify SR^43^ and only value their function or utility, which suggests that the distinction between the two is not entirely clear. Future research could investigate this question, which would advance the field of the economy of action as well as SBT.

Finally, several limitations of our study should be considered. First, to the best of our knowledge, our study is the first to show a link between Extraversion and the perceived benefits of tools. Therefore, our results should be viewed with caution, and more research is needed to explain the causal mechanisms underlying this relationship. Second, the comparison between the tool and the SR was made with independent samples, which prevents a strong comparison. Third, the sample of Experiment 1 was exclusively composed of women. This prevents any generalization of our results for both sexes. However, it is important to note that it was a methodological choice aimed to neutralize potential influence of Sex on the use of the SR^26–28^.

## Supplementary Information

Supplementary Information.

## References

[1] Proffitt DR (2006). Embodied perception and the economy of action. Perspect. Psychol. Sci..

[2] Schmid-Hempel P, Kacelnik A, Houston AI (1985). Honeybees maximize efficiency by not filling their crop. Behav. Ecol. Sociobiol..

[3] Kacelnik A, Houston AI, Schmid-Hempel P (1986). Central-place foraging in honey bees: the effect of travel time and nectar flow on crop filling. Behav. Ecol. Sociobiol..

[4] Davies NB, Krebs JR, West SA (2012). An Introduction to Behavioural Ecology.

[5] Sparrow WA, Newell KM (1998). Metabolic energy expenditure and the regulation of movement economy. Psychon. Bull. Rev..

[6] Bitgood S, Dukes S (2006). Not another step! economy of movement and pedestrian choice point behavior in shopping malls. Environ. Behav..

[7] Bhalla M, Proffitt DR (1999). Visual-motor recalibration in geographical slant perception. J. Exp. Psychol. Hum. Percept. Perform..

[8] Witt JK, Proffitt DR, Epstein W (2004). Perceiving distance: a role of effort and intent. Perception.

[9] Schnall S, Zadra JR, Proffitt DR (2010). Direct evidence for the economy of action: glucose and the perception of geographical slant. Perception.

[10] Witt JK, Sugovic M (2013). Spiders appear to move faster than non-threatening objects regardless of one’s ability to block them. Acta Psychol. (Amst.).

[11] Gross, E. B. & Proffitt, D. The economy of social resources and its influence on spatial perceptions. *Front. Hum. Neurosci.* **7**, 772 (2013). 10.3389/fnhum.2013.00772 PMC3832788 24312039

[12] Doerrfeld A, Sebanz N, Shiffrar M (2012). Expecting to lift a box together makes the load look lighter. Psychol. Res..

[13] Schnall S, Harber KD, Stefanucci JK, Proffitt DR (2008). Social support and the perception of geographical slant. J. Exp. Soc. Psychol..

[14] Beckes L, Coan JA (2011). Social baseline theory: the role of social proximity in emotion and economy of action: social baseline theory. Soc. Personal. Psychol. Compass.

[15] Coan JA, Sbarra DA (2015). Social baseline theory: the social regulation of risk and effort. Curr. Opin. Psychol..

[16] Gross EB, Medina-DeVilliers SE (2020). Cognitive processes unfold in a social context: a review and extension of social baseline theory. Front. Psychol..

[17] Asendorpf JB, Wilpers S (1998). Personality effects on social relationships. J. Pers. Soc. Psychol..

[18] Von Dras DD, Siegler IC (1997). Stability in extraversion and aspects of social support at midlife. J. Pers. Soc. Psychol..

[19] Srivastava S, Angelo KM, Vallereux SR (2008). Extraversion and positive affect: a day reconstruction study of person–environment transactions. J. Res. Personal..

[20] Stephan Y, Boiché J, Canada B, Terracciano A (2014). Association of personality with physical, social, and mental activities across the lifespan: findings from US and French samples. Br. J. Psychol..

[21] Swickert RJ, Rosentreter CJ, Hittner JB, Mushrush JE (2002). Extraversion, social support processes, and stress. Personal. Individ. Differ..

[22] Halamandaris KF, Power KG (1999). Individual differences, social support and coping with the examination stress: a study of the psychosocial and academic adjustment of first year home students. Personal. Individ. Differ..

[23] Amirkhan JH, Risinger RT, Swickert RJ (1995). Extraversion: a ‘hidden’ personality factor in coping?. J. Pers..

[24] Swickert RJ, Hittner JB, Foster A (2010). Big Five traits interact to predict perceived social support. Personal. Individ. Differ..

[25] Osiurak F, Morgado N, Vallet GT, Drot M, Palluel-Germain R (2014). Getting a tool gives wings: overestimation of tool-related benefits in a motor imagery task and a decision task. Psychol. Res..

[26] Reevy GM, Maslach C (2001). Use of social support: gender and personality differences. Sex Roles.

[27] Fiske ST (2018). Social Cognition: Selected Works of Susan Fiske.

[28] Saad G, Gill T (2001). Sex differences in the ultimatum game: an evolutionary psychology perspective. J. Bioeconomics.

[29] Faul F, Erdfelder E, Buchner A, Lang A-G (2009). Statistical power analyses using G*Power 3.1: tests for correlation and regression analyses. Behav. Res. Methods.

[30] Mathôt S, Schreij D, Theeuwes J (2012). OpenSesame: an open-source, graphical experiment builder for the social sciences. Behav. Res. Methods.

[31] John, O. P., Donahue, E. M. & Kentle, R. L. Big Five Inventory-Versions 4a and 54 (Institute of Personality and Social Research, 1991).

[32] Plaisant O, Courtois R, Réveillère C, Mendelsohn GA, John OP (2010). Validation par analyse factorielle du Big Five Inventory français (BFI-Fr). Analyse convergente avec le NEO-PI-R. Ann. Méd. Psychol. Rev. Psychiatr..

[33] McCrae, R. R. & Costa Jr, P. T. The five-factor theory of personality. in *Handbook of Personality: Theory and Research* (eds. John, O. P., Robins, R. W. & Pervin, L. A.) 159–181 (Guilford Press, 2008).

[34] Zchaluk K, Foster DH (2009). Model-free estimation of the psychometric function. Atten. Percept. Psychophys..

[35] Lakens D, McLatchie N, Isager PM, Scheel AM, Dienes Z (2020). Improving inferences about null effects with Bayes factors and equivalence tests. J. Gerontol. Ser. B.

[36] Thompson ER (2008). Development and validation of an international English big-five mini-markers. Personal. Individ. Differ..

[37] Goldenberg G, Iriki A (2007). From sticks to coffee-maker: mastery of tools and technology by human and non-human primates. Cortex.

[38] Witt JK, Proffitt DR (2008). Action-specific influences on distance perception: a role for motor simulation. J. Exp. Psychol. Hum. Percept. Perform..

[39] Depue RA, Collins PF (1999). Neurobiology of the structure of personality: dopamine, facilitation of incentive motivation, and extraversion. Behav. Brain Sci..

[40] Lucas RE, Diener E, Grob A, Suh EM, Shao L (2000). Cross-cultural evidence for the fundamental features of extraversion. J. Pers. Soc. Psychol..

[41] Coan JA, Schaefer HS, Davidson RJ (2006). Lending a hand: social regulation of the neural response to threat. Psychol. Sci..

[42] Belk, R. Alternative conceptualizations of the extended self. *Adv Consum Res,* **42**, 251–254 (2014).

[43] LaCroix JM, Pratto F (2015). Instrumentality and the denial of personhood: the social psychology of objectifying others. Rev. Int. Psychol. Soc..

