# Supplementary material for: Extraversion level predicts perceived benefits from social resources and tool use
Source: Sci Rep. 2021 Jun 10;11:12260. doi: 10.1038/s41598-021-91298-w (PMC8192896; doi:10.1038/s41598-021-91298-w)
Supplement: Supplementary file 1 — Supplementary Information. [file 41598_2021_91298_MOESM1_ESM.docx]

**Extraversion level predicts perceived benefits from social resources and tool use**

Vincent MURDAY^1^*, Kévin Campos-Moinier^1^, François Osiurak^2^, Lionel Brunel^1^

^1^Laboratoire Epsylon, Université Montpellier 3, Université Montpellier, FRANCE

^2^Laboratoire EMC, Université Lyon 2, Lyon, France

Correspondence concerning this article should be addressed to Vincent Murday, Laboratoire Epsylon EA 4556, 4bd Henri IV, 34000 Montpellier, FRANCE. Email: [vincent.murday@gmail.com](mailto:vincent.murday@gmail.com)

## Supplementary Material

Supplementary Material concerns the decision times for each quantity of rolls in each experiment. We added these analyzes in order to further differentiate the perceived benefits between the tool and the social resource.

Experiment 1

A one-way ANOVA was run on Decision times with Quantity of rolls (Q4 vs. Q8 vs. Q12 vs. Q16 vs. Q20 vs. Q24) as a within-subjects factor. We found a significant main effect, *F*(5, 175) = 25.21, *p* < .001, $\eta_{\rho}^{2}$= .419. More specifically, participants took more time to decide about Q12 than Q4 (*t*(35) = 7,538, *p* < .001), Q8 (*t*(35) = 3.496, *p* = .001), Q16 (*t*(35) = 2.436, *p* = .02), Q20 (*t*(35) = 6.069, *p* < .001), and Q24 (*t*(35) = 7.432, *p* < .001). Decision times were also longer for Q8 than for Q4 (*t*(35) = 6.506, *p* < .001), Q20, *t*(35) = 2.981, *p* = .005) and Q24 (*t*(35) = 5.594, *p* < .001), Q16 than Q4 (*t*(35) = 5.131, *p* < .001), Q20 (*t*(35) = 6.012, *p*< .001), and Q24 (*t*(35) = 6.473, *p* < .001), and longer for Q20 than for Q24 (*t*(35) = 3.151, *p* = .003). Moreover, no differences were found between Q4 and Q20 (*t*(35) = 1.803, *p* = .08), Q24 (*t*(35) = 0.278, *p*= .783), and between Q16 and Q8 (*t*(35) = 0.906, *p* = .371). Results are shown in Supplementary Figure S1.

Supplementary Figure S1 – Decision time as a function of the quantity of rolls (Experiment 1). Error bars indicate standard errors to the means.

Experiment 2

A one-way ANOVA was run on Decision times with Quantity of rolls (Q4 vs. Q8 vs. Q12 vs. Q16 vs. Q20 vs. Q24) as a within-subjects factor. We found a significant main effect (*F*(5, 175) = 13.24, *p*< 0.001, $\eta_{\rho}^{2}$ = 0.274). More specifically, participants took more time to decide about Q12 than Q4 (*t*(35) = 3.505, *p* = .001), Q16 (*t*(35) = 3.237, *p* = .003), Q20 (*t*(35) = 4.655, *p* < .001), and Q24 (*t*(35) = 5.134, *p* < .001). Decision times were also longer for Q8 than for Q4 (*t(35)* = 4.049, *p* < .001), Q20 (*t*(35) = 4.908, *p*< .001), Q24 (*t*(35) = 6.406, *p* < .001), Q16 than for Q20 (*t*(35) = 3.921, *p* < .001) and Q24 (*t*(35) = 4,110, *p* < .001). Moreover, no differences were found between Q12 and Q8 (t(35) = 1.742, *p* = .090), Q4 and Q20 (*t*(35) = .531, *p* = .599), Q24 (*t*(35) = 1.77, *p* = .085), Q8 and Q16 (*t*(35) = 0.815, *p* = .421), Q20 and Q24 (*t*(35) = 1.712 *p*= .096). Results are shown in Supplementary Figure S2.

Supplementary Figure S2 – Decision times as a function of the quantity of rolls (Experiment 2). Error bars indicate standard errors to the means.

Experiments 1 and 2

A two-way ANOVA was run on Decision times, with Quantity of rolls (Q4 vs. Q8 vs. Q12 vs. Q16 vs. Q20 vs. Q24) as a within-subjects factor and Experiment (Social vs. Tool) as a between-subjects factor. A significant main effect was found for Quantity of rolls, *F*(5, 350) = 35.904, *p* < .001, $\eta_{\rho}^{2}$ = .335. However, we found no main effect of Experiment, *F*(1, 70) = .327, *p* = .569 and no interaction effect between Experiment and Quantity of rolls, *F*(5, 350) = 1.132, *p* = .343. Results are shown in Supplementary Figure S3.

Supplementary Figure S3 – Decision times as a function of quantity of rolls for SR and the Tool
